# A two-component system serves as a central hub for connecting energy metabolism and plasmid dissemination in bacteria

**DOI:** 10.1128/mbio.02474-23

**Published:** 2023-11-30

**Authors:** Tomoko Kubori

**Affiliations:** 1 Department of Microbiology, Graduate School of Medicine, Gifu University, Gifu, Japan; University of Illinois Chicago, Chicago, Illinois, USA

**Keywords:** Plasmid conjugation, type IV secretion system, Dot/Icm, antibiotic resistance, GacA/S two component system, bacterial metabolism, *Legionella pneumophila*, *Acinetobacter baumannii*

## Abstract

Mobile genetic elements such as conjugative plasmids play a key role in the acquisition of antibiotic resistance by pathogenic bacteria. Resistance genes on plasmids can be transferred between bacteria using specialized conjugation machinery. *Acinetobacter baumannii*, the most common bacterium associated with nosocomial infections, harbors a large conjugative plasmid that encodes a type IV secretion system (T4SS). Feng et al. recently found that the *A. baumannii* T4SS is specialized for plasmid transfer, suggesting that it may be involved in multidrug resistance (Z. Feng, L. Wang, Q. Guan, X. Chu, and Z.-Q. Luo*,* mBio e02276-23, 2023, https://doi.org/10.1128/mbio.02276-23), T4SS-encoding genes are shown to be controlled by a versatile GacA/S two-component regulatory system. GacA/S is also found to regulate genes involved in central metabolism. The coordinated regulation of metabolism and plasmid conjugation may be a bacterial strategy for adapting to selective pressure from antibiotics.

## COMMENTARY

Hospital-acquired infections with opportunistic microorganisms are a global public health threat. Bacterial pathogens in the genus *Acinetobacter* are commonly involved in nosocomial infections. The gram-negative bacterium *Acinetobacter baumannii* is widely associated with multidrug resistance (MDR), which poses a serious challenge to clinical treatment (1). *A. baumannii* harbors a large, self-transmissible plasmid and small mobilizable plasmids. The large conjugative plasmid (LCP) encodes genes required for MDR (2), promoting the spread of infection. Feng et al. (3) found that the *A. baumannii* type IV secretion system (T4SS) encoded on the LCP plays an important role in plasmid transfer.

**Fig 1:**
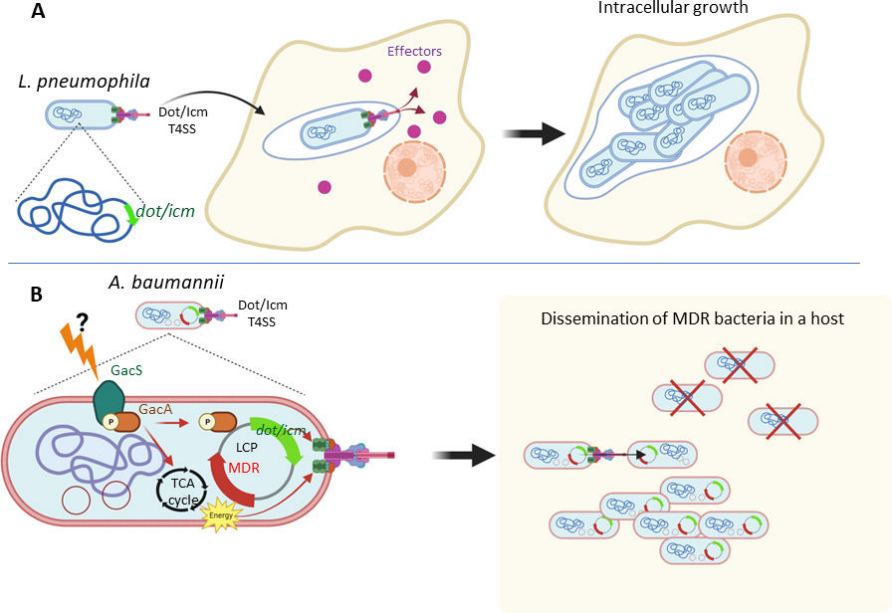
Roles of Dot/Icm T4SSs during *L. pneumophila* (**A**) and *A. baumannii* (**B**) infection. (**A**) The *L. pneumophila* Dot/Icm system translocates a large array of effector proteins into the host cell cytosol. The orchestrated enzymatic activities of these proteins modify host cell systems and enable bacteria to build a replicative niche. (**B**) The *A. baumannii* Dot/Icm system appears to function as plasmid transfer machinery. The system is encoded on the LCP and is transcriptionally regulated by the GacA/S two-component system. In response to unidentified environmental signals, GacA/S upregulates the expression of the *dot/icm* genes, enhancing the efficiency of plasmid transfer. As a versatile regulator, GacA/S also controls the expression of genes involved in the tricarboxylic acid (TCA) cycle. The coordinated regulation of bacterial metabolism and plasmid transfer mediated by the Dot/Icm system is thought to optimize the dissemination of bacteria harboring plasmids that encode MDR genes. This illustration was created using BioRender.com.

T4SSs are key players in bacterial pathogenicity. They include diverse translocation machinery consisting of two subfamilies: (i) DNA conjugation systems and (ii) bacterial protein translocators. *Legionella pneumophila*, a causative agent of acute pneumonia, Legionnaires’ disease, possesses *dot/icm* genes in two separate loci that encode component proteins of a T4SS essential for the translocation of bacterial effector proteins into the host cell cytosol (4) (Fig. 1). *L. pneumophila* Dot/Icm proteins have limited but significant sequence similarities to conjugation systems such as the *Agrobacterium* VirB/D4 and *Escherichia coli* plasmid R388 (4–6). While the *L. pneumophila* Dot/Icm system can transfer both proteins and DNA substrates (7, 8), however, the biological significance of the latter remains unknown.

Genetic studies have revealed that genes on the *A. baumannii* LCPs encode protein orthologs of *L. pneumophila* Dot/Icm proteins (9, 10). In contrast to the *L. pneumophila* Dot/Icm system, however, the *A. baumannii* (Ab) Dot/Icm system functions as conjugation machinery, aiding in the dissemination of the LCP and mobilizable plasmids (3). Since the LCP carries genes required for antibiotic resistance, the Dot/Icm T4SS is thought to play an important role in pathogen spread by mediating the transfer of the MDR plasmid. Ab *dot/icm* gene expression is regulated by the GacA/S two-component system (TCS; 3), indicating that this system is needed to promote the efficient transfer of plasmids via the Ab T4SS (Fig. 1).

TCSs control diverse biological processes required to reprogram microbial physiology in response to environmental cues. Multiple TCSs are involved in regulating the virulence of *L. pneumophila* (11), of which PmrA/B (12, 13) and CpxR/A (14, 15) function as direct regulators of *dot/icm* genes and Dot/Icm translocated effector proteins. While the *L. pneumophila* LetA/S, an ortholog of GacA/S, impacts several bacterial traits (16), its involvement in regulating Dot/Icm T4SS remains unknown.

Signaling networks involving the GacA/S TCS have been extensively assessed in *Pseudomonas* (17). GacA/S globally impacts the *Pseudomonas aeruginosa* transcriptome and metabolome, significantly affecting the transcription of about 15% of its genes (18). GacA/S also regulates the production of secondary metabolites in various bacteria (17) and the expression of various genes including those encoding key metabolic enzymes. A recent study found that nutrient compounds, such as succinate, fumarate, malate, and oxaloacetate, can induce the expression of Ab *dot/icm* genes even if they have no effect on bacterial growth (3). The GacA/S system plays a role in regulating the expression of genes associated with metabolic pathways in which succinate and malate are involved (3). Using the larvae of *Galleria mellonella*, a model organism for evaluating infection with various bacterial pathogens including *P. aeruginosa* (19), the GacA/S system was shown to be required for *A. baumannii* virulence (3).

The involvement of TCA cycle intermediates in the induction of *A. baumannii* metabolic genes indicates that a positive circuit is likely involved in increasing nutrient conditions in these bacteria. The Dot/Icm system is composed of more than 20 proteins. Both constructing the system architecture and transferring substrates are energy-consuming processes (20). The co-regulation of *dot/icm* and metabolic genes by the TCS could be a strategy by which *A. baumannii* organizes its bacterial systems. Metabolically favorable conditions are likely required for *A. baumannii* to disseminate the MDR plasmid and assure its proliferation in the presence of competing bacteria.

Interestingly, the type VI secretion system (T6SS) encoded by the *A. baumannii* chromosome limits LCP dissemination through conjugation (10). This is shown by the ability of T6SSs to kill neighboring bacteria, thereby preventing them from being recipients of plasmid conjugation. To aid successful conjugation, the LCP encodes TetR transcriptional regulators that suppress the T6SS of the Ab host (2). Ab T4SS-mediated regulation of plasmid dissemination restricts T6SS activity and ensures successful bacterial spread and the acquisition of antibiotic resistance.

The signal that activates the GacA/S system to induce the Ab T4SS has not yet been identified. In general, nutrient conditions and other environmental states define bacterial behavior using TCSs. GacA/S TCS is a global regulator of bacterial physiology, including the production of secondary metabolites, regulation of secretion systems, regulation of bacterial motility, and production of quorum-sensing molecules (17). Antibiotic resistance is acquired through communication between bacteria that reside in complex environments, such as human organs, to which they need to adapt. Future studies are required to define whether and how the Ab T4SS impacts bacterial virulence in environments in which they are exposed to antibiotics.

Research by Feng et al. has expanded our knowledge of the biological roles of the Dot/Icm T4SS and provided a novel example of the fine-tuned networks of bacterial physiology and virulence on which TCSs act as central hubs. Further study of the mechanisms by which bacterial conjugation systems are regulated could inform the development of treatments that target and manipulate these systems.
